# Exploring the Association Between AI-Derived Competency Scores and Nurse Retention

**DOI:** 10.3390/healthcare14172755

**Published:** 2026-08-31

**Authors:** Young Hye Song, Hyoung Ran Park, Mi Ran Kim, Hyeongju Ryu

**Affiliations:** 1 Department of Nursing, Konkuk University Medical Center, Seoul 05030, Republic of Korea; yhsong@kuh.ac.kr (Y.H.S.);; 2 Department of Nursing and Research Institute for Biomedical & Health Science, Konkuk University, Chungju 27478, Republic of Korea

**Keywords:** artificial intelligence, AI-based recruitment, nurse retention, employee turnover, competency assessment, newly employed nurses, healthcare workforce

## Abstract

Background: This study explored the associations between artificial intelligence (AI)-derived competency scores and nurse retention outcomes among newly employed nurses. Evidence regarding the relationship between AI-based competency assessments, nurse performance, and long-term retention remains limited. Methods: Data were collected from 156 newly employed nurses at hospitals in South Korea between March 2021 and August 2023. Retention status (retained vs. resigned) was specified as the dependent variable, while AI-derived competency scores served as independent variables in binary logistic regression analyses. Result: Self-reflection was significantly associated with retention (*p* = 0.032), whereas honesty (*p* = 0.051) and values (*p* = 0.072) did not achieve statistical significance and were therefore not associated with retention. Behavioral response was significantly associated with early turnover (*p* = 0.035), whereas relationship (*p* = 0.073) and understanding of others (*p* = 0.141) were not significantly associated with turnover. Conclusions: These findings provide preliminary evidence that selected AI-derived competency scores are associated with nurse retention outcomes. However, the findings should be interpreted cautiously because this study examined statistical associations rather than predictive performance, and further validation studies are required before AI-assisted competency assessments can be considered standalone decision-support tools for nurse recruitment.

## 1. Introduction

The recruitment and retention of nursing staff in healthcare institutions are critical for ensuring patient safety and stable operations [1,2]. In South Korea, the demand for nurses is high, as the number of hospital beds is three times the Organization for Economic Co-operation and Development average [3]. However, securing sufficient personnel is increasingly challenging due to structural issues such as high work intensity, difficulties in adapting to clinical environments, and high turnover rates [4,5,6]. These challenges negatively affect both hospital operations and nursing service quality [7].

To address this shortage, the South Korean government and related organizations have increased nursing college enrolments, and approximately 20,000 nurses are produced annually [8]. Despite this increase, clinical nursing shortages persist because many graduates fail to remain in the field long-term [6,9].

In South Korea, a national nursing licensing exam is held annually for graduates. Based on this exam, major hospitals conduct large-scale recruitment [10]. Approximately 20,000 nurses are hired annually, but many struggle to adapt and resign early. In 2022, 57.4% of new nurses resigned, with 34.8% leaving within the first year due to work adaptation issues [9]. High turnover increases workloads, lowers job satisfaction, reduces care quality, and raises recruitment costs [11].

Advancements in technology have introduced artificial intelligence (AI)-based competency assessments in recruitment. Many employers use AI tools to identify top talent and streamline recruitment processes [12,13]. In healthcare, AI has been proposed as a tool that may improve the efficiency of recruitment processes and support hiring decisions in some settings [14]. However, evidence regarding its ability to improve recruitment outcomes in healthcare remains limited. AI is especially advantageous for processing large application volumes [12], and many South Korean hospitals use AI competency assessments for large-scale nurse recruitment [10].

Among the AI-based recruitment systems used in South Korea, the competency assessment developed by MIDAS IN has been adopted by numerous public and private organizations. The system infers applicants’ competency profiles using AI-assisted analysis of game-based assessment data and behavioral responses. The underlying technology is protected by a Korean patent entitled *System and Methods for Evaluating and Deducing Interviewer’s Ability to Use Interview Game* (Korean Patent No. 10-2205955) [15,16]. Recent evidence also suggests that AI-assisted personnel assessment may provide meaningful information regarding interpersonal competencies in healthcare settings, although independent validation studies remain limited. Accordingly, further empirical evidence is needed to evaluate whether competency scores generated by such systems are associated with actual workforce outcomes, including nurse retention and early turnover.

Although evidence regarding the relationship between AI-based recruitment, nurse performance, and long-term retention remains limited, AI-assisted competency assessments have been increasingly adopted in nurse recruitment [17]. This study examined the associations between AI-derived competency assessment scores and retention duration among newly employed nurses. Specifically, we sought to identify competency domains associated with nurse retention and early turnover. Furthermore, we explored the potential value and limitations of AI-assisted competency assessments in nursing recruitment and identified directions for future validation studies.

A preliminary, short abstract-style report of this pilot dataset was previously presented at a conference and archived as a brief, two-page proceeding [18]. This comprehensive, full-length manuscript substantially expands upon that initial work by presenting a systematic, hierarchical analysis of the competency framework across three distinct levels (upper, middle, and lower), providing a deeply elaborated theoretical discussion on the psychological and organizational mechanisms behind the findings, and introducing concrete operational safeguards and clinical implications for nursing administration.

## 2. Methods

### 2.1. Data Collection

This study was based on data collected and archived between 1 March 2021 and 31 August 2023, including AI competency assessment results and retention durations of new nurse recruits.

### 2.2. Study Population and Data Selection

This secondary data analysis used the full dataset stored at the institution to maximize statistical power, including interview results and employment durations for 156 nurses who joined through open recruitment between 2021 and 2022. Participants were classified into two groups according to their employment duration during the first year after appointment. Early turnover was defined as employment for less than 365 days, whereas retention was defined as employment for 365 days or longer.

**Figure 1:**
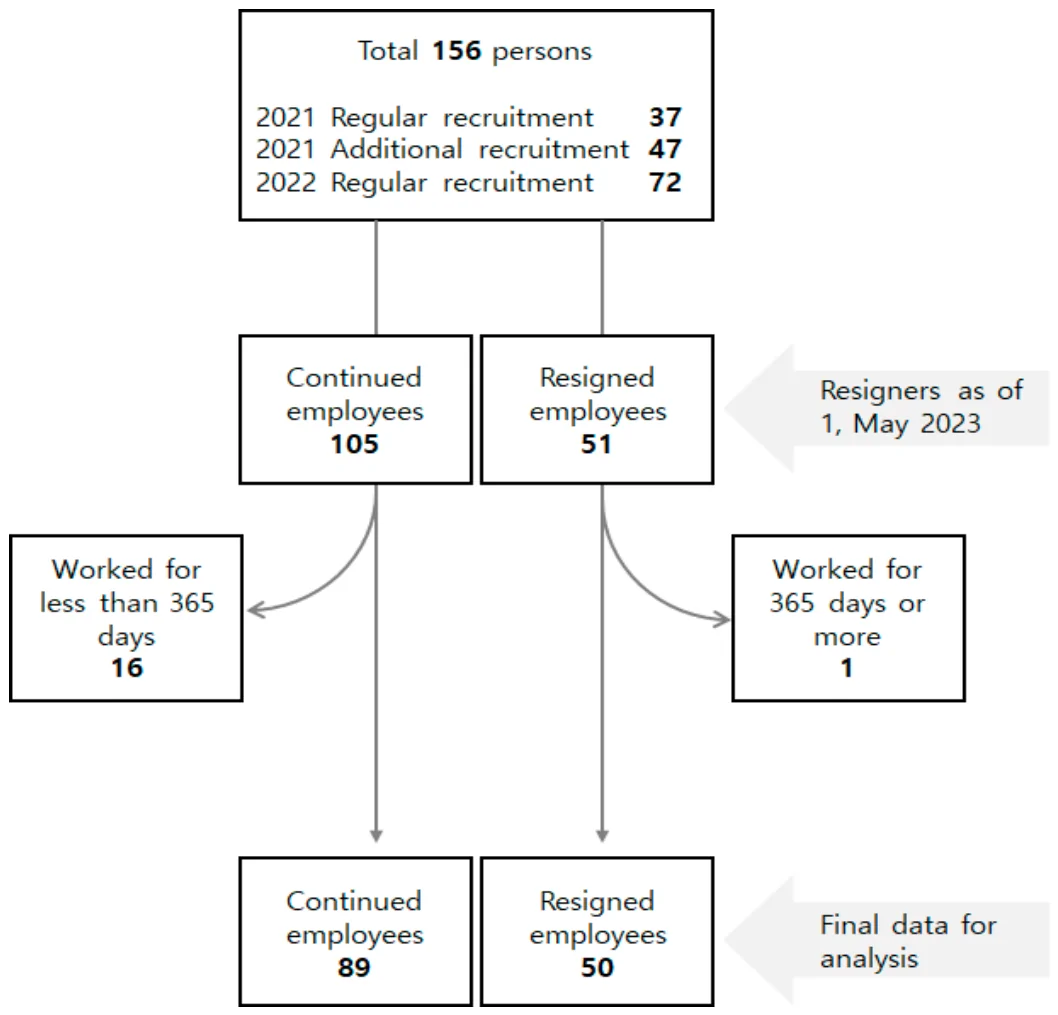
Study participant selection and data collection process.

Based on the institutional employment records, 105 participants were initially classified as retained and 51 as resigned. However, these administrative classifications were not fully consistent with the predefined study outcome criteria. Retention was defined as employment for 365 days or longer, whereas early turnover was defined as employment for less than 365 days. Accordingly, 16 participants initially classified as retained had employment durations of less than 365 days, and one participant initially classified as resigned had remained employed for 365 days or longer. These 17 cases were excluded to ensure consistent outcome classification based on the predefined 365-day threshold. The final analytic sample therefore consisted of 89 retained nurses and 50 early leavers, for a total of 139 participants, as illustrated in Figure 1.

### 2.3. Research Design and Analysis

This study employed a secondary analysis of archived administrative data. Descriptive statistics and participant characteristics were analyzed using SPSS version 27.0 (IBM Corp., Armonk, NY, USA). Binary logistic regression analyses were conducted to examine the associations between AI-derived competency scores and retention outcomes.

The AI competency assessment provides competency scores at three hierarchical levels (upper-, middle-, and lower-level competencies). To reflect this hierarchical structure, three separate binary logistic regression models were constructed for the upper-, middle-, and lower-level competencies. Each model examined the associations between competency scores at the corresponding hierarchical level and retention status. Because competency scores across the hierarchical framework are conceptually related, separate regression models were constructed for each level to facilitate interpretation while avoiding the inclusion of conceptually overlapping competency variables within a single model.

Within each model, all competency variables belonging to the corresponding hierarchical level were entered simultaneously using the enter method. No variable selection procedure was performed because previous evidence identifying specific AI-derived competency domains associated with nurse retention remains limited. Therefore, all variables within each competency level were included to explore their potential associations with retention outcomes.

Retention status (retention vs. turnover) was specified as the dependent variable, whereas competency scores at the corresponding hierarchical level were entered as independent variables.

### 2.4. Data Structure and Characteristics

The AI competency assessment used in this study was developed by MIDAS IN (Seongnam, Republic of Korea) as a commercial recruitment support system. The assessment utilizes AI-assisted analysis of game-based behavioral responses to infer applicants’ competency profiles and is protected by a Korean patent [15]. The assessment results are organized into a hierarchical framework consisting of 59 competency items, including seven upper-level competencies, 15 middle-level competencies, and 37 lower-level competencies. Middle-level competency scores are derived from their corresponding lower-level competencies, while upper-level competencies are generated from the middle-level competency scores. Each competency score was treated as an independent AI-derived variable in the present study, and detailed conceptual definitions are provided in Appendix A.

Because the assessment is a proprietary commercial system, detailed information regarding the underlying AI algorithm, model training procedures, feature weighting, and psychometric validation was not available to the researchers. Therefore, the present study focused on evaluating the associations between the AI-generated competency scores and nurse retention outcomes rather than validating the assessment algorithm itself.

### 2.5. Ethical Considerations

This study adhered to the latest revision of the Declaration of Helsinki and the International Council for Harmonisation Good Clinical Practice guidelines. Ethical review and approval were waived by the Institutional Review Board of Konkuk University Medical Center (KUMC 2023-11-008) due to the retrospective use of de-identified data. Data collection and analysis complied with these ethical standards, and all study data were securely stored in password-protected files under the responsibility of the principal investigator in a locked research facility.

## 3. Results

Descriptive statistical analyses comparing the retention and turnover groups are presented in Table 1. The average employment duration for the retention group was 490.11 days (±68.84), significantly longer than the turnover group’s average of 94.38 days (±83.13). The analysis systematically evaluated the structured competency scores across the AI assessment’s hierarchical framework, specifically analyzing the 7 upper-level, 15 middle-level, and 37 lower-level competency variables in separate models.

The analysis revealed notable differences between the retention and turnover groups in terms of AI competency assessment variables. In the retention group, the variables with the highest mean scores were emotional perception (59.20 ± 11.84), negativity (56.55 ± 9.94), depression (56.05 ± 9.98), simultaneous processing (57.07 ± 10.43), and communication (56.40 ± 7.87). Conversely, the variables with the lowest mean scores were achievement seeking (43.80 ± 9.15), proactivity (47.15 ± 6.79), growth seeking (47.98 ± 11.76), change management (48.06 ± 9.39), and behavioral response (48.48 ± 9.66).

In the turnover group, the variables with the highest mean scores were emotional perception (61.43 ± 11.19), negativity (58.15 ± 10.03), depression (57.45 ± 10.49), simultaneous processing (56.88 ± 10.74), and hostility (56.16 ± 9.49). The lowest-scoring variables in the turnover group included achievement seeking (44.91 ± 11.18), proactivity (48.62 ± 8.62), honesty (48.70 ± 10.58), change management (49.06 ± 10.40), and self-reflection (49.37 ± 7.48).

Both groups exhibited high scores in emotional perception, simultaneous processing, negativity, and depression, while consistently low scores were observed in achievement seeking, proactivity, and change management.

Because no prior evidence clearly identified which AI-derived competency variables are associated with nurse retention, all variables within each hierarchical competency level were simultaneously entered into their respective logistic regression models using the enter method. Binary logistic regression analysis was conducted at each hierarchical level of the AI competency assessment. Table 2 presents the regression analysis results, highlighting variables with significant influence on nurse retention and early turnover.

For the middle-level competencies, self-reflection was identified as a statistically significant factor (*p* = 0.032). Self-reflection refers to the ability to objectively understand oneself with a humble attitude. For every one-unit increase in self-reflection, the likelihood of retention increased by approximately 7.2%.

Among the upper-level competencies, no upper-level competency was significantly associated with retention outcomes.

Among the lower-level competencies, behavioral response emerged as a significant predictor (*p* = 0.035). Behavioral response refers to the ability to communicate appropriately with others to achieve performance goals. A one-unit increase in behavioral response was associated with an approximately 8.4% lower likelihood of retention. These findings suggest that the role of behavioral response in nurse retention warrants further investigation.

Although honesty did not reach statistical significance (*p* = 0.051), a one-unit increase in honesty score showed only a marginal, non-significant trend (estimated 4.7% higher likelihood of retention). Because this relationship strictly failed to meet the significance threshold, it cannot be inferred as a reliable factor. Honesty reflects a moral attitude characterized by truthfulness, adherence to principles, and consistency between words and actions.

Relationship (OR = 0.926, 95% CI = 0.852–1.007, *p* = 0.073) and understanding of others (OR = 0.948, 95% CI = 0.884–1.018, *p* = 0.141) showed only modest estimated effect sizes and were strictly non-significant, suggesting no statistical association with the outcomes within this sample.

## 4. Discussion

This study used binary logistic regression analyses to examine the associations between AI-derived competency scores and nurse retention outcomes among newly employed nurses. Self-reflection was positively associated with retention, whereas behavioral response was significantly associated with early turnover. Although these findings provide preliminary evidence of associations between AI-derived competency scores and nurse retention outcomes, they should be interpreted cautiously and require replication in larger independent samples.

### 4.1. Variables Positively Influencing Retention

Self-reflection emerged as a significant predictor of retention, as it encompasses self-regulation and awareness. Nurses with strong self-reflection can effectively manage their emotions and behaviors, even in challenging situations, fostering positive attitudes and loyalty toward their organizations. Jeon and Lee found that self-efficacy and resilience, key components of self-reflection, positively influence retention intentions by helping nurses adapt to demanding environments [19]. Reflective practices also improve job satisfaction and commitment [20].

Although honesty has been reported to support organizational trust and collaboration in previous studies [21,22], it did not achieve statistical significance in the present study (*p* = 0.051). To maintain statistical conservatism, we refrain from drawing practical conclusions regarding honesty’s role in nurse retention from our sample, classifying it strictly as non-significant.

Similarly, values were not significantly associated with retention in this study despite previous evidence suggesting their importance in organizational commitment [23,24].

### 4.2. Variables Positively Influencing Early Turnover

Higher behavioral response scores were associated with an increased likelihood of early turnover in the present study. This finding was unexpected because behavioral response is conceptually defined as the ability to communicate and interact effectively with others to achieve work-related goals. Rather than indicating a direct causal relationship, this unexpected association may reflect specific characteristics of the assessment tool, unmeasured organizational factors, or other confounding variables. Because the underlying algorithm and weighting processes of this proprietary system are not publicly disclosed, it remains unclear which specific behavioral features drove this competency domain. Indeed, this lack of transparency highlights a critical limitation of relying on commercial ‘black-box’ AI competency assessments in clinical settings; because there are no clear mechanistic clues to explain how or why these AI-derived scores relate to actual nursing retention, the tool’s practical utility remains constrained by this explainability gap. Consequently, further replication studies using more transparent, open-access competency metrics are required to clarify these nuances. Future studies should examine whether similar findings can be reproduced using alternative competency assessment instruments or more transparent AI-based evaluation methods.

It is also possible that behavioral response interacts with organizational rather than individual factors. Previous studies have shown that excessive stress among Generation Z novice nurses contributes to turnover intentions, while lower resilience reduces the ability to cope with workplace stress and increases turnover risk [17,25]. Organizational support deficiencies, excessive workload, and stressful early work environments have also been consistently identified as important contributors to early turnover among newly graduated nurses [26,27]. Because these organizational variables were not measured in the present study, this alternative explanation could not be empirically evaluated. It is also plausible that individuals with stronger behavioral response competencies may possess higher interpersonal awareness and greater expectations regarding communication, teamwork, and organizational support. When these expectations are not met in demanding clinical environments, such individuals may be more likely to experience dissatisfaction and voluntarily leave the organization. Although this interpretation is speculative, it provides one possible theoretical explanation for the unexpected association observed in the present study.

Although relationship competency and understanding of others were not significantly associated with turnover in the present study, previous studies have suggested that interpersonal stress, emotional exhaustion, and workplace conflict are important contributors to early turnover among novice nurses [28,29,30,31]. Because these competencies strictly failed to reach statistical significance, any conclusions regarding their independent influence on turnover are avoided to prevent extending beyond what the data support. Nevertheless, these variables remain non-significant trends that may warrant prospective investigation in larger and more diverse samples.

### 4.3. Clinical and Practical Implications for Nursing Management

The findings of this study offer several actionable insights for nurse managers and healthcare administrators seeking to optimize recruitment and early onboarding processes. First, AI-derived competency scores should be treated strictly as supplementary, decision-support information rather than standalone screening or rejection thresholds. Because these metrics capture broad behavioral tendencies, they are best utilized to complement traditional, face-to-face structured interviews. For example, if a candidate exhibits a high behavioral response score—which was associated with early turnover in our sample—interviewers can use this information as a prompt to ask targeted, behavioral questions during the interview to probe the candidate’s realistic job expectations and coping mechanisms under pressure.

Second, healthcare organizations must implement robust institutional safeguards when adopting proprietary AI assessment tools. Nursing administrations should establish review committees to oversee AI implementation, ensuring that automated algorithms do not introduce systematic bias or replace professional human judgment in hiring.

Lastly, these competency profiles can be used post-hire to design personalized transition-to-practice programs. For instance, newly graduated nurses showing lower self-reflection scores could be assigned to structured reflective practice groups or paired with experienced mentors to foster emotional resilience and clinical adaptation during their critical first year of practice.

### 4.4. Limitations and Recommendations

Despite its novel insights, this study has several limitations that must be acknowledged. First, the retrospective secondary analysis was conducted using data from a single institution with a final sample of 139 participants. This limited sample size poses a key methodological constraint when evaluated against the relatively large number of independent variables entered into our regression models, particularly in the lower-level competency model, which analyzed 37 variables simultaneously. Such a high variable-to-sample ratio significantly increases the risk of model overfitting and reduces statistical power, which may compromise the stability and reproducibility of the regression estimates. Furthermore, due to the reliance on administrative records, we were unable to control for crucial unmeasured organizational variables known to influence nurse retention, such as leadership style, ward workload, staffing ratios, organizational support, and overall job satisfaction. Consequently, the observed associations between AI scores and turnover should be understood within the context of these unmeasured environmental influences.

Second, the commercial AI assessment system (MIDAS IN) utilized in this study is proprietary. As a result, detailed information regarding its underlying algorithmic architecture, training datasets, feature weights, and psychometric validation was inaccessible to the researchers. Therefore, this study serves strictly to evaluate statistical associations with clinical retention outcomes rather than validating the technical performance of the proprietary AI algorithm itself. Given the single-center design and exploratory nature of our analysis, these findings represent preliminary evidence rather than definitive proof of predictive utility, and they should not be extrapolated as evidence that the AI tool can accurately predict nurse retention in other settings.

To position this exploratory work as a preliminary foundation, future research directions must be significantly expanded. First, prospective studies utilizing multicenter cohorts are required to facilitate rigorous external validation of these findings across diverse clinical settings. Second, future researchers should build and evaluate prospective prediction models, explicitly reporting predictive performance measures such as sensitivity, specificity, and Area Under the Curve (AUC). Third, direct comparisons with traditional structured interview assessments should be conducted to establish the incremental validity of AI tools. Finally, comprehensive psychometric evaluations of the AI-derived competency framework are warranted to ensure its construct validity and reliability within the unique, high-stakes environment of clinical nursing.

## 5. Conclusions

This study explored the associations between AI-derived competency scores and nurse retention outcomes among newly employed clinical nurses. Our exploratory analysis revealed that self-reflection was positively associated with retention, whereas behavioral response was significantly associated with early turnover. These findings suggest that specific AI-derived competency domains may serve as useful supplementary benchmarks for understanding nurse retention. However, these associations represent preliminary exploratory evidence requiring prospective external validation before they can inform actual recruitment practices.

Furthermore, the integration of AI-assisted competency assessments in healthcare recruitment raises critical ethical and operational considerations. Due to the limited transparency of proprietary algorithms, concerns regarding explainability, fairness, and potential algorithmic bias must be addressed. Therefore, AI-derived competency assessments should be implemented strictly as decision-support tools to complement, rather than replace, human professional judgment and structured face-to-face interviews. Crucially, competency scores generated by proprietary AI systems must not be used as a standalone criterion in recruitment decisions until independent, peer-reviewed evidence supporting their predictive validity becomes available. Ultimately, a balanced approach that combines data-driven insights with robust organizational support and reflective practices holds the greatest promise for improving clinical nurse retention and fostering a sustainable healthcare workforce.

## Figures and Tables

**Table 1:** Descriptive statistics of AI-derived competency scores according to the hierarchical competency structure.

		Retained Group (N = 89)				Early Leavers (N = 50)			
		Min	Max	Mean	SD	Min	Max	Mean	SD
Employment Length *		365.00	607.00	490.11	68.84	0.00	364.00	94.38	83.13
**AI variable**									
**Upper-level competencies**	**Middle-Level Competencies**								
Reliability	Positivity	29.09	76.60	53.20	8.93	31.70	74.55	55.01	8.97
	Proactivity	31.18	66.23	47.15	6.79	27.74	64.81	48.62	8.62
Strategy	Strategic thinking ability	27.21	67.96	54.21	7.27	33.08	67.10	54.14	6.63
	Strategic planning ability	23.93	73.96	55.28	9.03	24.34	70.09	55.78	8.31
Relationship	Understanding of others	34.33	71.64	52.59	6.95	34.13	75.33	54.58	8.89
	Relationship responsiveness	26.41	71.54	51.51	8.36	28.27	74.51	53.12	9.61
Execution	Adaptability	30.68	64.16	50.13	6.70	36.12	71.77	50.80	7.78
	Perseverance	33.32	76.83	53.30	8.79	26.73	77.34	54.37	10.94
Values	Empathy	34.16	73.97	52.78	8.56	37.16	78.72	53.34	10.12
	Self-reflection	35.98	72.22	50.58	7.27	33.29	69.85	49.37	7.48
Organizational Fit	Neuroticism	32.00	74.07	53.72	8.71	38.86	72.33	54.78	8.84
	Negative Emotionality	40.00	75.02	54.37	7.74	35.95	75.02	56.10	9.21
Affinity	Interview attitude	31.62	67.00	52.94	8.05	33.59	67.00	52.44	8.45
	Communication skills	36.48	67.00	54.80	8.52	33.25	67.00	53.49	7.34
	Interpersonal attraction	28.24	67.00	55.22	8.99	35.43	67.00	54.47	8.24
**AI variable**									
**Middle-level competencies**	**Lower-Level Competencies**								
Positivity	Self-affirmation	22.79	80.00	52.74	9.95	32.01	75.77	54.66	10.97
	Environmental positivity	31.74	78.47	53.56	9.68	31.50	75.91	55.25	9.61
Positivity	Reward seeking	20.00	77.40	50.33	8.55	27.38	68.90	50.04	8.04
	Growth seeking	20.00	78.54	47.98	11.76	20.00	78.54	50.49	12.71
	Achievement seeking	20.00	63.17	43.80	9.15	20.00	64.02	44.91	11.18
Strategic thinking ability	Reasoning ability	20.00	73.16	52.93	10.44	20.00	68.02	51.44	9.52
	Learning ability	34.16	76.10	54.45	8.56	38.05	71.83	55.40	8.17
	Planning ability	21.99	75.44	52.78	12.20	31.13	75.44	53.39	9.92
Strategic planning ability	Information processing	20.00	80.00	54.33	13.11	20.00	80.00	55.91	11.97
	Simultaneous processing	20.00	75.35	57.07	10.43	20.00	74.32	56.88	10.74
	Cognitive control	26.43	75.40	55.11	9.55	25.76	75.05	55.00	9.30
Understanding of others	Emotional perception	27.59	80.00	59.20	11.84	34.61	80.00	61.43	11.19
	Intention perception	20.00	74.81	49.84	9.41	29.91	74.81	51.68	10.87
Relationship responsiveness	Behavioral response	20.00	71.70	48.48	9.66	24.55	74.48	51.67	10.38
	Emotional response	27.12	74.52	52.92	8.97	30.11	74.52	53.81	10.66
Adaptability	Change management	20.00	66.74	48.06	9.39	25.88	78.43	49.06	10.40
	Risk management	35.48	68.19	51.45	7.66	34.80	65.30	51.88	8.45
Perseverance	Goal orientation	31.10	75.61	51.07	9.81	26.10	75.61	53.07	11.52
	Conscientiousness	26.24	78.04	54.56	10.08	20.00	78.04	54.98	11.63
Empathy	Cooperativeness	25.64	79.99	53.61	10.25	21.62	79.99	54.79	11.98
	Respectfulness	28.23	72.45	53.23	9.79	31.39	77.88	52.47	11.31
	Responsibility	35.15	78.59	51.10	8.87	34.62	78.59	52.96	9.85
Self-reflection	Objectivity	25.74	73.45	48.64	9.66	27.58	73.45	50.24	11.19
	Humility	24.58	80.00	51.44	12.17	25.95	80.00	50.57	12.35
	Honesty	28.14	78.52	51.12	10.56	25.32	69.21	48.70	10.58
Neuroticism	Anxiety	25.51	80.00	52.42	9.64	28.78	74.60	53.42	9.50
	Depression	30.99	76.04	56.05	9.98	36.27	76.04	57.45	10.49
	Stress	32.01	75.83	53.80	9.95	32.76	75.83	54.68	10.46
Negative Emotionality	Hostility	36.09	69.82	53.92	8.72	37.94	69.82	56.16	9.49
	Negativity	36.48	78.18	56.55	9.94	39.77	78.18	58.15	10.03
	Impulsiveness	33.57	74.47	52.87	9.29	26.81	74.47	54.58	11.38
Interview attitude	Stability	34.82	67.00	51.79	7.92	36.01	67.00	51.65	8.95
	Self-confidence	30.80	67.00	53.85	9.19	28.41	67.00	52.47	8.10
Communication skills	Communication	34.08	67.00	56.40	7.87	29.84	67.00	55.31	7.29
	Emotional expression	28.31	67.00	53.21	9.67	33.04	67.00	51.85	8.34
Interpersonal attraction	Attractiveness	31.97	67.00	55.66	9.01	31.81	67.00	54.91	8.38
	Trustworthiness	26.24	67.00	54.31	8.95	38.01	67.00	53.43	8.46

* As of 1 May 2023. Competencies are presented according to the hierarchical structure of the AI competency assessment (upper, middle, and lower levels).

**Table 2:** Binary logistic regression results between long-tenured nurses and early leavers.

	B	SE	Wald	*p*-Value	Exp (B)	95% CI Lower	95% CI Upper	VIF
Upper-level competencies								
Reliability	−0.029	0.041	0.496	0.481	0.972	0.897	1.053	2.255
Strategy	0.012	0.034	0.129	0.719	1.012	0.947	1.082	1.416
Relationship	−0.077	0.043	3.224	0.073	0.926	0.852	1.007	2.684
Execution	−0.004	0.038	0.011	0.916	0.996	0.925	1.073	1.817
Values	0.083	0.046	3.244	0.072	1.087	0.993	1.190	2.853
Organizational Fit	−0.017	0.040	0.175	0.676	0.983	0.909	1.064	2.976
Affinity	0.029	0.024	1.487	0.223	1.029	0.983	1.078	1.164
Middle-level competencies								
Positivity	−0.020	0.036	0.326	0.568	0.980	0.913	1.051	2.940
Proactivity	−0.034	0.036	0.892	0.345	0.966	0.900	1.038	2.182
Strategic thinking ability	0.003	0.031	0.008	0.930	1.003	0.943	1.066	1.412
Strategic planning ability	0.001	0.027	0.000	0.984	1.001	0.949	1.055	1.619
Understanding of others	−0.053	0.036	2.166	0.141	0.948	0.884	1.018	2.098
Relationship responsiveness	0.019	0.044	0.183	0.669	1.019	0.934	1.112	4.354
Adaptability	−0.013	0.030	0.190	0.663	0.987	0.930	1.047	1.385
Perseverance	−0.002	0.029	0.004	0.947	0.998	0.943	1.057	2.335
Empathy	0.040	0.037	1.157	0.282	1.041	0.968	1.119	3.349
Self-reflection	0.070	0.032	4.616	0.032	1.072	1.006	1.143	1.499
Neuroticism	0.003	0.038	0.008	0.928	1.003	0.932	1.080	3.322
Negative Emotionality	−0.053	0.042	1.539	0.215	0.949	0.873	1.031	3.696
Interview attitude	−0.043	0.050	0.729	0.393	0.958	0.868	1.057	4.552
Communication skills	0.085	0.064	1.775	0.183	1.088	0.961	1.233	7.526
Interpersonal attraction	−0.011	0.056	0.037	0.848	0.989	0.887	1.104	6.932
Lower-Level Competencies								
Self-affirmation	0.032	0.040	0.630	0.427	1.033	0.954	1.118	4.379
Environmental positivity	−0.016	0.037	0.190	0.663	0.984	0.914	1.059	3.639
Reward seeking	0.006	0.026	0.054	0.815	1.006	0.955	1.060	1.254
Growth seeking	−0.025	0.031	0.626	0.429	0.976	0.918	1.037	3.635
Achievement seeking	−0.011	0.033	0.102	0.749	0.989	0.927	1.056	2.662
Reasoning ability	0.038	0.026	2.210	0.137	1.039	0.988	1.093	1.717
Learning ability	0.013	0.027	0.242	0.623	1.013	0.961	1.069	1.343
Planning ability	−0.015	0.022	0.440	0.507	0.985	0.944	1.029	1.643
Information processing	−0.005	0.020	0.059	0.808	0.995	0.957	1.034	1.720
Simultaneous processing	0.016	0.027	0.359	0.549	1.016	0.965	1.070	2.031
Cognitive control	−0.005	0.030	0.028	0.867	0.995	0.937	1.056	2.169
Emotional perception	−0.022	0.022	0.951	0.329	0.978	0.936	1.022	1.778
Intention perception	−0.007	0.042	0.032	0.858	0.993	0.915	1.077	4.191
Behavioral response	−0.087	0.041	4.463	0.035	0.916	0.845	0.994	3.647
Emotional response	0.074	0.050	2.214	0.137	1.077	0.977	1.187	5.701
Change management	−0.004	0.027	0.020	0.887	0.996	0.945	1.050	1.870
Risk management	−0.017	0.033	0.276	0.600	0.983	0.922	1.048	1.791
Goal orientation	−0.029	0.046	0.394	0.530	0.971	0.887	1.064	5.796
Conscientiousness	0.004	0.031	0.016	0.898	1.004	0.945	1.067	2.993
Cooperativeness	0.013	0.036	0.129	0.720	1.013	0.943	1.088	3.862
Respectfulness	0.047	0.034	1.920	0.166	1.048	0.981	1.120	3.234
Responsibility	−0.019	0.045	0.177	0.674	0.981	0.898	1.072	3.980
Objectivity	0.028	0.032	0.750	0.387	1.028	0.966	1.094	2.921
Humility	0.009	0.022	0.185	0.667	1.009	0.967	1.053	1.830
Honesty	0.046	0.023	3.802	0.051	1.047	1.000	1.096	1.591
Anxiety	−0.033	0.038	0.773	0.379	0.967	0.899	1.042	3.502
Depression	0.023	0.036	0.410	0.522	1.023	0.954	1.098	3.649
Stress	0.033	0.040	0.671	0.413	1.033	0.956	1.117	4.332
Hostility	−0.052	0.041	1.670	0.196	0.949	0.876	1.027	3.550
Negativity	−0.020	0.044	0.205	0.651	0.980	0.900	1.068	4.624
Impulsiveness	−0.041	0.046	0.778	0.378	0.960	0.878	1.051	5.307
Stability	0.003	0.036	0.008	0.928	1.003	0.934	1.077	2.434
Self-confidence	−0.009	0.066	0.017	0.897	0.991	0.871	1.129	9.185
Communication	0.022	0.054	0.166	0.684	1.022	0.919	1.137	4.581
Emotional expression	0.032	0.066	0.237	0.627	1.032	0.908	1.174	9.880
Attractiveness	0.013	0.080	0.028	0.868	1.013	0.867	1.184	12.929
Trustworthiness	−0.009	0.063	0.019	0.891	0.991	0.876	1.122	7.736

## Data Availability

The data presented in this study are not publicly available due to privacy and ethical restrictions.

## Abbreviations

The following abbreviations are used in this manuscript:

AI	Artificial Intelligence
KUMC	Konkuk University Medical Center
SPSS	Statistical Package for the Social Sciences

## Appendix A

**Table A1:** AI variable composition concept.

Upper-Level Competencies	Middle-Level Competencies	Lower-Level Competencies	Composition Concept
**Reliability**	Confidence and a positive attitude that motivate individuals to achieve performance goals.		
	**Positivity**	A progressive attitude based on positive beliefs about oneself and the environment.	
		**Self-affirmation**	Acting autonomously with confidence and belief in one’s abilities.
		**Environmental positivity**	Embracing changes in the environment positively and striving to make improvements.
	**Proactivity**	A strong desire and will for personal growth and greater achievements.	
		**Reward seeking**	Sensitivity and pursuit of rewards.
		**Growth seeking**	A desire to enhance one’s abilities based on intrinsic motivation.
		**Achievement seeking**	The attitude of striving for social accomplishments and success.
**Strategy**	The ability to control internal and external variables to effectively establish strategies.		
	**Strategic thinking ability**	The ability to analyze fixed variables related to problem-solving and establish effective strategies.	
		**Reasoning ability**	The ability to compare and analyze the values of situations or concepts.
		**Learning ability**	The ability to quickly acquire new information and apply it to different situations.
		**Planning ability**	The ability to create specific plans for goal achievement through simulation.
	**Strategic planning ability**	The ability to handle dynamic variables in problem-solving processes with a goal-oriented approach.	
		**Information processing**	The ability to absorb new information and apply it to existing knowledge.
		**Simultaneous processing**	The ability to quickly switch attention between different tasks and handle them simultaneously.
		**Cognitive control**	The ability to suppress unnecessary information or distractions and selectively focus on relevant goals.
**Relationship**	The ability to understand and respond appropriately to others to manage interpersonal variables.		
	**Understanding of others**	The ability to accurately grasp the emotions and intentions of others.	
		**Emotional perception**	The ability to accurately perceive the emotions of others.
		**Intention perception**	The ability to understand the background and intentions behind others’ words or actions.
	**Relationship responsiveness**	Managing one’s emotions and responding appropriately to interpersonal situations.	
		**Behavioral response**	The ability to communicate and act appropriately with others to achieve performance goals.
		**Emotional response**	Managing one’s emotions during tasks and responding appropriately to others’ emotions.
**Execution**	The ability to persistently execute actions and respond to variables during the achievement process.		
	**Adaptability**	The ability to quickly identify and adapt to changes or risks during the execution process.	
		**Change management**	The ability to manage rapidly changing information simultaneously.
		**Risk management**	The ability to detect risks by gathering information and formulate appropriate responses.
	**Perseverance**	The ability to persistently sustain actions for achieving goals.	
		**Goal orientation**	Continuously monitoring actions to align with performance objectives and adjusting through feedback.
		**Conscientiousness**	Demonstrating consistent and persistent behavior to achieve goals.
**Values**	The capacity to fulfill social responsibilities based on self-awareness and empathy.		
	**Empathy**	Understanding the social value of empathy and cooperating with others responsibly.	
		**Cooperativeness**	Accepting others’ perspectives, cooperating, and maintaining harmonious relationships.
		**Respectfulness**	Respecting others’ individuality and showing such behavior.
		**Responsibility**	Recognizing one’s influence on others and taking responsibility for one’s actions.
	**Self-reflection**	The ability to reflect on oneself with humility and objective understanding.	
		**Objectivity**	Recognizing one’s inner self objectively and accepting others’ perspectives on oneself.
		**Humility**	Reflecting on one’s abilities without assuming superiority over others.
		**Honesty**	Acting truthfully, ethically, and morally without deceit or pretense.
**Organizational Fit**	Psychological or behavioral traits that may hinder organizational synergy.		
	**Neuroticism**	Excessive psychological tension and anxiety in daily or stressful situations.	
		**Anxiety**	A tendency to worry easily over minor issues.
		**Depression**	A state of low motivation and lack of vitality in general.
		**Stress**	Emotional strain that persists even after some time has passed since experiencing a stressful event.
	**Negative Emotionality**	Expressions of negative emotions towards others or society that undermine organizational synergy.	
		**Hostility**	A tendency to distrust others’ intentions and exhibit psychological aggression.
		**Negativity**	A tendency to perceive society pessimistically without valid justification.
		**Impulsiveness**	A lack of control over emotions and behaviors, leading to impulsive actions.
Affinity	The ability to deliver favorable impressions and decisions through situational communication.		
	**Interview attitude**	Emotional stability and confidence demonstrated during interviews.	
		**Stability**	A stable demeanor without anxiety or nervousness during interviews.
		**Self-confidence**	A demeanor of confidence and assertiveness in actions or speech.
	**Communication skills**	The ability to convey emotions and intentions clearly and vividly.	
		**Communication**	The ability to articulate one’s message clearly to others.
		**Emotional expression**	The ability to appropriately express emotions or sentiments in different situations.
	**Interpersonal attraction**	The ability to project likability and trustworthiness through appropriate language, expressions, and gestures.	
		**Attractiveness**	The degree to which one projects a favorable impression on others.
		**Trustworthiness**	The degree to which one inspires trust in others.
